# Apoptotic and Nonapoptotic Activities of Pterostilbene against Cancer

**DOI:** 10.3390/ijms19010287

**Published:** 2018-01-18

**Authors:** Rong-Jane Chen, Hsiao-Che Kuo, Li-Hsin Cheng, Yu-Hsuan Lee, Wen-Tsan Chang, Bour-Jr Wang, Ying-Jan Wang, Hung-Chi Cheng

**Affiliations:** 1 Department of Food Safety/Hygiene and Risk Management, College of Medicine, National Cheng Kung University, Tainan 70101, Taiwan; janekhc@gmail.com (R.-J.C.); bmm175@hotmail.com (Y.-H.L.); 2 Department of Biochemistry and Molecular Biology, College of Medicine, National Cheng-Kung University, Tainan 70101, Taiwan; shoujer@gmail.com (H.-C.K.); wtchang@mail.ncku.edu.tw (W.-T.C.); 3 The Institute of Basic Medical Sciences, College of Medicine, National Cheng Kung University, Tainan 70101, Taiwan; lindsie0419@gmail.com; 4 Department of Environmental and Occupational Health, College of Medicine, National Cheng Kung University, Tainan 70101, Taiwan; pochih.wang@msa.hinet.net; 5 Department of Occupational and Environmental Medicine, National Cheng Kung University Hospital, Tainan 70101, Taiwan; 6 Department of Cosmetic Science and Institute of Cosmetic Science, Chia Nan University of Pharmacy and Science, Tainan 707010, Taiwan; 7 Graduate Institute of Clinical Medicine, College of Medicine, Taipei Medical University, Taipei 11031, Taiwan; 8 Department of Medical Research, China Medical University Hospital, China Medical University, Taichung 40401, Taiwan; 9 Department of Biomedical Informatics, Asia University, Taichung 41354, Taiwan

**Keywords:** apoptosis, fibronectin, cancer metastasis, pterostilbene

## Abstract

Cancer is a major cause of death. The outcomes of current therapeutic strategies against cancer often ironically lead to even increased mortality due to the subsequent drug resistance and to metastatic recurrence. Alternative medicines are thus urgently needed. Cumulative evidence has pointed out that pterostilbene (*trans*-3,5-dimethoxy-4-hydroxystilbene, PS) has excellent pharmacological benefits for the prevention and treatment for various types of cancer in their different stages of progression by evoking apoptotic or nonapoptotic anti-cancer activities. In this review article, we first update current knowledge regarding tumor progression toward accomplishment of metastasis. Subsequently, we review current literature regarding the anti-cancer activities of PS. Finally, we provide future perspectives to clinically utilize PS as novel cancer therapeutic remedies. We, therefore, conclude and propose that PS is one ideal alternative medicine to be administered in the diet as a nutritional supplement.

## 1. Introduction

Cancer is one of major causes of death. It has been estimated that around 13.2 million cancer patients will die yearly by 2030 worldwide [1]. Over the past 25 years, efforts in understanding how tumor cells are transformed from normal cells have led to discoveries of clinically important tumor-targeting drugs as cancer therapeutics [2]. However, the outcomes of these therapeutic strategies, mainly exerting pro-apoptotic effects on cancer cells, often ironically cause increased mortality due to the subsequent drug resistance and to metastatic recurrence [3]. Therefore, increasing attention has been paid to complementary and alternative medicines that take advantages of the medicinal and functional dietary herbs or phytochemicals to concertedly fight against cancers [4]. Cumulative evidence has pointed out that pterostilbene (*trans*-3,5-dimethoxy-4-hydroxystilbene, PS), a dimethylated analog of resveratrol [5], has excellent pharmacological benefits for the prevention and treatment for various types of cancer by evoking apoptosis or apoptosis-independent effects [6]. In this review article, we will update current knowledge regarding tumor progression toward accomplishment of metastasis, on which PS has apoptosis-dependent and -independent effects. Finally, we will provide future perspectives to clinically utilize PS as novel cancer therapeutic remedies.

## 2. Tumor Progression, Circulating Tumor Cells, and Metastasis

**Figure 1:**
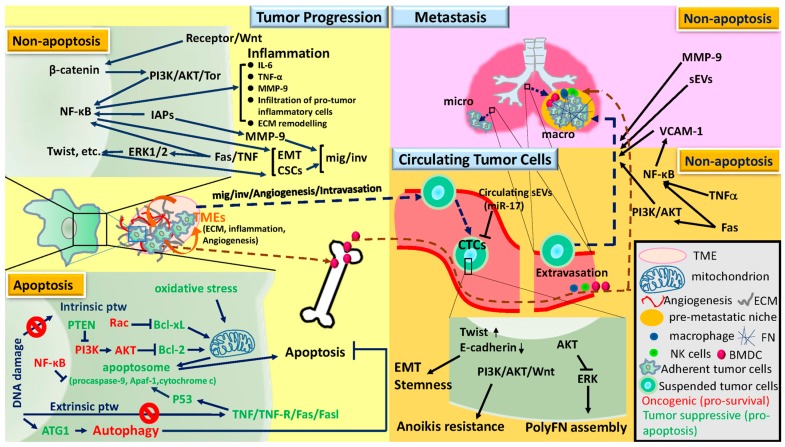
Genes and pathways involved in the tumor progression toward cancer metastasis. Metastasis is the final stage of a chronic tumor progression, starting from tumor transformation followed by early progression within the primary tumor tissues, blood-borne to become circulating tumor cells (CTCs), colonization into distant organs in which pre-metastatic niche has been built, and outgrowth as secondary tumor tissues. Listed are signaling pathways associated with intrinsic and extrinsic stimulations for apoptosis and with apoptosis-independent activities. Note: The genes marked in green are considered tumor suppressive and in red oncogenic. The arrows with broken lines indicate the directions and locations toward which particular cells are moving. The corresponding full names abbreviated are listed as follows: TMEs, tumor microenvironments; ECM, extracellular matrix; micro, micrometastasis; macro, macrometastasis; FN, fibronectin; BMDC, bone marrow derived cells; TNF, tumor necrosis factor family, ptw, pathway; mig, migration; inv, invasion.

Metastasis is the final stage of a chronic process for tumor cells through signaling pathways associated with apoptosis or independent of apoptosis to develop. It is starting from tumor transformation, followed by early progression within the primary tumor tissues, blood-borne to become circulating tumor cells (CTCs), colonization into distant organs in which pre-metastatic niche has been built, and outgrowth as secondary tumor tissues [7,8,9,10]. Here, we will focus and review current understandings only on the molecular mechanisms underlying tumor progression toward metastasis in distant organs (Figure 1), on which PS is likely to have effects. For deeper and detailed knowledge regarding cancer metastasis, readers should direct to other review articles [11,12,13].

### 2.1. Tumor Progression

During tumor progression, tumor cells are able to conquer the lethal threatening from DNA damage caused by diverse carcinogens due to the bypass of pre-toxic lesions by autophagy and inhibition of apoptosis [10]. Apoptosis is typically dysregulated in human cancers by pro-survival factors, e.g., nuclear factor-κB (NF-κB), and AKT, and by pro-apoptosis components, e.g., Fas ligand (FASL) and p53-upregulated modulator of apoptosis (PUMA) [10,14]. The apoptotic-signaling cascade is possibly mediated either by the extrinsic pathway triggered by the ligation between soluble molecules such as the tumor necrosis factor (TNF) family and cell surface death receptors (DR) including members of the TNF-receptor (TNF-R) family [14,15,16]) or the intrinsic pathway triggered by various mitochondrial stimuli such as oxidative stress and depletion of growth factor [17] to form apoptosome, which is composed of procaspase-9, Apaf-1, and cytochrome c [18]. These mechanisms also underlie the effects of microenvironments on tumor transformation and progression [19,20]. Consistently, either inhibitions of pro-apoptotic or activations of pro-survival factors in tumor cells contribute to the functions of many oncogenes and tumor suppressor genes (TSGs) in promoting tumor transformation and progression. For examples, the oncogene Rac-1 [21] decreases the phosphorylation levels of pro-apoptosis protein Bcl-xL through intrinsic pathway [22]. Conversely, the TSG PTEN inactivates PI3K to reduce AKT [23], leading to inactivation of anti-apoptotic B cell lymphoma-2 (Bcl-2) protein in the intrinsic pathway [24]. Moreover, DNA damage often activates autophagy by activating ATG1, a principal initiator of autophagy [25]. It has been reported that autophagy promotes tumor progression and thereby resistance to cancer therapy [26]. Accordingly, autophagy inhibitors may provide benefits to synergize with anticancer drugs [26]. However, one should use caution to apply autophagy inhibitors in cancer therapy in that autophagy has conversely been proposed as an anti-cancer cellular event [26].

After the consequential genomic instability yielded from activation of oncogenes and inactivation of TSGs renders cells resistant to apoptosis, tumor microenvironments (TMEs) join and concertedly drive tumor cells to evolve in an apoptotic resistance-independent manner [27,28]. Cell migration and invasion mediated by epithelial mesenchymal transition (EMT) and matrix metalloproteinase-9 (MMP-9) within TMEs are prerequisite for tumor cells to disseminate from primary tissues [29,30]. Although Fas, a member of TNF receptor superfamily, is critically involved in triggering extrinsic apoptotic signals in susceptible cells [31], accumulating evidence indicates the participation of Fas activation in various nonapoptotic pathways during tumorigenesis and tumor progression [32]. It has indeed been reported that Fas signaling activated-ERK1/2 is able to trigger EMT and invasion in glioblastoma cancer cells in vivo, leading to eminent metastasis [33]. Similarly, given that TNF is the main ligand to trigger the extrinsic apoptotic pathway, the alternative role that TNF plays is to stimulate pro-tumor inflammation through upregulation of NF-κB that induces the expression of various pro-inflammatory genes and also participates in inflammasome regulation [34,35]. Interestingly, the anti-apoptotic inhibitors of apoptosis proteins (IAPs) have also been recognized to function independently of apoptosis-resistance as E3 ubiquitin ligases and regulators of NF-κB signaling, upregulating MMP-9 and EMT-related transcription factors and promoting tumor migration, invasion, and metastasis [14,36,37]. In addition, cancer stem cells (CSCs) have long been reported to be an essential element responsible for the malignant tumor progression and anti-cancer drug resistance through EMT [37,38]. Wnt/β-catenin-mediated signaling is able to foster CSC development through NF-κB activation that is involved in EMT-associated transcriptional regulation [39] by engaging the PI3K-Akt-TOR pathway [40]. CSCs often acquire resistance to apoptosis-inducing anticancer drugs. For example, defects in the death receptor pathway often seen in CSCs, favoring the apoptotic resistance, IAP overexpression, and aberrant activation of NF-κB [41].

### 2.2. Circulating Tumor Cells

Anoikis is induced when normal cells, mostly epithelial-origin, are detached from the supporting extracellular matrix (ECM) and become homeless cells, in which either the intrinsic or extrinsic pathway directing apoptosis are often increased [42,43]. PI3K-AKT signaling axis has been identified as the driving force for homeless tumor cells to survive anoikis and intravasate to become circulating tumor cells (CTCs) [44]. In mouse pancreatic CTCs, noncanonical WNT signaling potentially contributes to the anoikis resistance [45]. Additionally, Twist, an essential element for EMT and cancer stemness, enables CTCs to survive in the circulation [8]. Strikingly consistent with these findings is the expression of fibronectin (FN), one of EMT markers, in both primary tumor cells and CTCs with a sustained AKT activity has experimentally and clinically been associated with high risk of cancer metastasis and poor prognosis and survival [46,47,48,49]. Alternative factors in promoting intravasation of tumor cells in primary organs into the circulation are exemplified by the stroma in TMEs that drives tumor cell migration and invasion [9]. Upon attaching to a path through the ECM, tumor cells migrate as single cells or collectively as a troop of cells in the tumor invasion front [50]. Furthermore, it has been reported that macrophages, endothelial cells, and invasive tumor cells considered as a triad are required for tumor intravasation [51]. Subsequently, given that natural killer (NK) cells are mainly responsible for the cytotoxicity of CTCs [52,53], neutrophils have been found to enhance metastasis formation via inactivating NK cell function through secretion of IL-1β and MMPs to significantly increase intraluminal survival time and facilitate transendothelial extravasation of CTCs [54].

Recently, polymeric assembly of FN (polyFN) on CTCs through a self-assembly process has been found to promote tumor cell colonization in the lungs independently of anoikis-resistance [48,49]. The ERK signaling pathway responsible for the polyFN assembly on CTCs is negatively regulated by AKT [49]. Indeed, the ERK1/2 pathway has emerged as a central regulator for the chemo-resistance of advanced cancer cells upon AKT-inactivation [55]. Clinically, FN expression has been identified as one of the prognostic biomarkers in CTCs of non-small cell lung cancer (NSCLC), gastric cancer, invasive breast cancer, and pancreatic cancer patients [45,56,57,58]. Clinically, CTCs, circulating small extracellular vesicles (sEVs), and cell-free microRNAs (miRs) in serum have been considered as excellent less invasive prognostic biomarkers for various types of cancer patients [59]. Exosomes are represented by a mixed population of sEVs that are smaller than 150 nm in diameter [60,61]. Various cargo-carrying sEVs can enter the circulation and likely exert effects on CTCs or TMEs including CTC-targeting NK cells and endothelia within distant organs to promote organ-specific metastasis [60,62]. It has been evidenced that sEVs incorporate and carry cell-free miRs to impact the functionalities of CTCs and NK cells [59]. The metastatic-suppressive roles of the circulating miR-17-92 cluster [63,64] have been manifested by the recruitment of M1 macrophage and NK cell infiltrations from the circulation [65]. miR-17, which might be enclosed in circulating sEVs [65], suppresses the transcription of endogenous FN in cells [66]. The fact that CTCs expressing high levels of polyFN assembly on cell surfaces are highly metastatic [48,49] is consistent with the finding that the circulating miR-17 is deemed as a metastatic suppressive miR [65].

### 2.3. Metastasis

At specific distant tissues, organ-preference extravasation follows when CTCs colonize the luminal walls of arterioles and capillaries through specific receptors [67]. Once extravasated, cancer cells may encounter local immunity and exist as dormant micrometastatic lesions for quite a long period of time until they properly adapt to the TMEs and become macrometastasis [7]. To establish macrometastasis, aberrant expression of NF-κB-dependent vascular cell adhesion molecule 1 (VCAM-1) promotes the transition from bone metastatic dormancy to overt metastasis [68]. Moreover, Fas may trigger pro-survival pathways through NF-κB [69] and PI3K/AKT [70] in apoptosis-resistant glioblastoma. In line with these findings, blockade of Fas signaling-induced cancer-related inflammation suppresses NF-κB-dependent breast tumor growth and macrometastasis [71]. Nevertheless, when the extravasated CTC-recruited macrophages provoke inflammation and act together with the pre-metastatic niche that has been built long before CTCs arrive, the appropriate TMEs facilitate macrometastatic growth of extravasated tumor cells in distant organs [72]. To facilitate distant tumor growth, sEVs carrying essential growth factors travel through the circulation to infiltrate distant organs by triggering vascular permeability [73] and to condition pre-metastatic sites [62]. Alternatively, pancreatic cancer cell-derived sEVs enter distant organs to build a pre-metastatic niche enriched in TGFβ, FN, and the chemokine that attracts macrophages [74].

In the pre-metastatic niche of distant organs, TNFα has been suggested to play a role in enhancing the invasion and metastatic growth of oral squamous cell carcinoma cells via the NF-κB in a pro-survival manner [75,76], whereas MMP-9 plays important roles in setting up a pre-metastatic niche for later extravasation of CTCs and the establishment of triple negative breast cancer metastasis [77,78]. Consistently, high serum levels of MMP-9 have been linked to metastasis and poor overall survival of cancer patients with melanoma [79]. Furthermore, expression of FN, secreted by fibroblasts, is upregulated in the pre-metastatic niche of secondary sites such as the lung, likely serving as a docking site for hematopoietic cells and the subsequent arrival of tumor cells [80]. A main reason for the pre-metastatic niche permissive to tumor colonization and macrometastatic growth is the recruitment of the main immunosuppressive myeloid-derived suppressor cells (MDSCs) [81] prior to the arrival of CTCs. After tumor cells arrive in the niche and overcome the inflammatory assaults, MDSCs further drive the infiltration of other immunosuppressive immune cells, e.g., M2 macrophages and N2 neutrophils to help establish macrometastasis [82].

## 3. PS Emerges as a Potent Alternative Medicine

PS, a dimethylated analog of resveratrol [5], was named after a natural phenolic compound found in *Pterocarpus marsupium* Roxb. (Fabaceae), which is native to India, Nepal, and Sri Lanka [83] and is one of the active compounds in the extracts of *P. marsupium* was used in Ayurvedic medicine for the treatment of diabetes. Now, we recognize this compound as PS, abundant also in blueberries [84]. Although PS and resveratrol can be found in almost the same food sources, PS appears to have higher bioavailability than resveratrol due to its dimethylether structure enhances lipophilicity and membrane permeability [6]. Substantial evidence suggests that PS may have diverse pharmacological benefits for the prevention and treatment for a vast range of human diseases, including cancer, dyslipidemia, diabetes, and cardiovascular and neurological degeneration [85]. The well-defined pharmacological actions of PS are antineoplastic, anti-inflammatory, and antioxidant [86]. Here, we will first focus on current knowledge regarding anti-inflammatory and antioxidant, leaving antineoplastic effects of PS alone in the next section.

PS is a well-recognized antioxidant with potent, concentration-dependent anti-oxidative effects [87,88]. PS reduces reactive oxygen species (ROS) and oxidative stress through the increased expression of the antioxidants catalase, total glutathione, glutathione peroxidase, glutathione reductase, and superoxide dismutase, which are implicated in the initiation and pathogenesis of several disease processes [86,89]. In addition to its possible modulation of antioxidant enzymes, PS also has an intrinsic antioxidant capacity that could be related to its diseases preventive effects [86]. Mechanistically, activation of the nuclear factor erythroid 2-related factor 2 (Nrf2) and its downstream target genes play a pivotal role in PS triggered antioxidant activity. Rapid activation of the Nrf2 pathway is important in preventing a variety of human diseases, including cancer, neurodegenerative and cardiovascular diseases, diabetes, and inflammatory diseases [90]. In one of our previous studies, we have demonstrated that PS is a more potent activator of Nrf2 signaling pathway than resveratrol, leading to inhibited cellular inflammation, high glucose-induced central nervous system injury, and oxidative stress, thereby preventing azoxymethane-induced colon carcinogenesis [91]. In addition, PS possesses the ability to protect the pancreatic β-cells against oxidative and cytokine stress in vitro, and improve β-cell function in a diabetes mice model in vivo via Nrf2 signaling cascade [92].

PS also targets diverse molecules involved in inflammatory responses, including inducible nitric oxide synthase (iNOS), cyclooxygenases (COX), leukotrienes, NF-κB, TNFα, and interleukins (ILs) for positive health effects against cancer, cardiovascular, and neurodegenerative diseases or diabetes [93]. Recent studies have shown that PS confers protection against myocardial ischemia/reperfusion (MI/R) injury. PS (10 mg/kg) dramatically improved cardiac function and reduced myocardial infarction following MI/R. The underlying mechanisms include the reductions of the expression of iNOS, p38 MAPK activation, and myocardial TNFα and IL-1β levels [94,95]. In an acute pancreatitis animal model, PS has been reported to reduce serum levels of TNFα/IL-1β and decrease the NF-κB gene expression in a dose-dependent manner, leading to attenuate the severe acute pancreatitis-induced tissue damage by decreasing the inflammatory response and protection of pancreatic tissues [96]. PS inhibits the activation of NF-κB by blocking the translocation of p65 to the nucleus and inactivating transcription factors, leading to the inactivation of COX-2 and iNOS [97].

## 4. Pro-Apoptotic Effects of PS against Cancer

**Figure 2:**
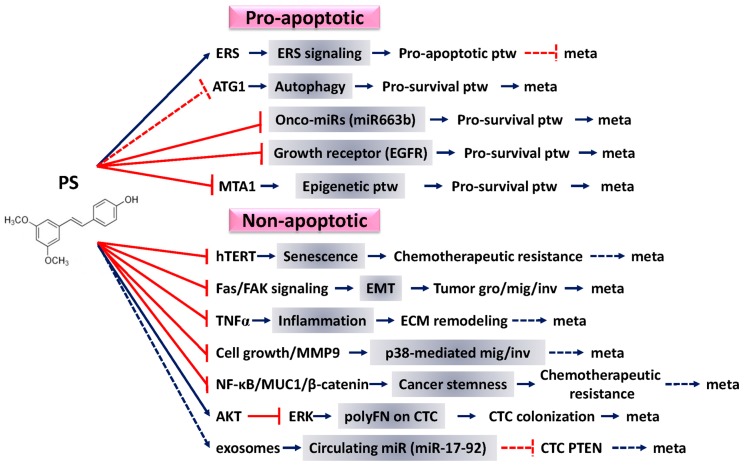
The overview of anticancer properties of pterostilbene (*trans*-3,5-dimethoxy-4-hydroxystilbene; PS). PS are able to target apoptosis-dependent and -independent signaling pathways against cancer progression and likely cancer metastasis. The key events affected by PS in all the pathways are boxed in grey color. In the category of pro-apoptotic effects, PS inhibits onco-miRs (e.g., miR663b) and growth factors (e.g., EGFR), triggers an epigenetic modification through the inhibition of MTA1, induces ERS, or lowers autophagy through the inhibition of ATG1. In the category of nonapoptotic effects, most of the effects caused by PS are shown to inhibit pro-tumor signaling pathways including hTERT, Fas/FAK signaling, TNFα, cell growth/MMP-9, NF-κB/MUC1/β-catenin, AKT/ERK signaling axis, and packed in circulating sEVs that are involved in senescence prevention, EMT, inflammation, p38/mediated migration/invasion, and cancer stemness, polyFN assembly on CTCs, and miR17-92-delivery in the circulation, respectively. All these apoptosis-dependent and -independent inhibitory effects caused by PS likely lead to a reduction of cancer metastasis. Note: signaling events involved in the anti-metastatic activities of PS are depicted as solid or broken lines whenever supported or not yet supported (only logically deduced), respectively, by the literature. The corresponding full names abbreviated are listed as follows: ptw, pathway; meta, metastasis; gro, growth; mig, migration; inv, invasion.

It has been estimated that only 0.01% of circulating tumor cells ultimately produce macro-metastases due in part to various stresses that cause cell death such as apoptosis [98]. Therefore, apoptosis induction could be one of the crucial strategies to treat metastatic cancers. Accordingly, one of the important anti-cancer effects PS is to facilitate both intrinsic and extrinsic apoptotic pathways [99]. Here, we review recent novel apoptosis-inducing mechanisms of PS (Figure 2) and summarize the pro-apoptotic effects of PS against cancers in Table 1.

### 4.1. Enhancement of Endoplasmic Reticulum Stress (ERS) Signaling

In NSCLC cell lines, PS enhances the ERS signaling by increasing the expression of ERS-related proteins (IRE-1, p-PERK, ATF4, and CHOP9), activating p53, and triggering the ERS-ROS signaling, that ultimately leads to apoptosis [29]. Similarly, PS may induce ERS, downregulate Bcl-2, upregulate pro-apoptosis related protein PUMA, and activate of caspases 9 and 12 in human esophageal cancer cells. In addition, PS also reduces tumor cell adhesion, migration, and intracellular GSG (glutathione) levels while increases the apoptosis index [100]. Consistently, PS significantly inhibits cell proliferation in chemosensitive and chemoresistant of bladder cancer cells through cell cycle arrest and apoptosis [101]. It has been reported that ROS generation plays an important role in the pro-apoptotic mechanism in PS-treated breast and prostate cancer cells, whereas PS increases antioxidant activity in pancreatic, esophageal, and colon cancer models but still exerts effective anticarcinogenic effects [91,102,103,104]. In vascular disease models, diabetes, and aging, the decrease of oxidative stress by PS is most likely acts as a protective role against disease process [116,117,118]. Thus, the relationship between PS and oxidation in cancer cell death is controversial and remains further investigation.

### 4.2. Induction of Autophagy

In addition to apoptosis, our previous studies have revealed a side effect of PS-induced protective autophagy for PS’s anti-cancer activities [26,101,119]. Thus, combined PS with autophagy inhibitor could enhance apoptosis in bladder cancer cells [101]. Echoing our results, Hsieh has also suggested that the combinatory strategy employing both PS and autophagy inhibitor strengthens the chemotherapeutic effect of PS in chemosensitive and chemoresistant lung cancer cells [105]. Nevertheless, it has been reported that PS suppresses cancer cell growth through apoptosis and autophagosome accumulation in various cancer cells, such as A375 melanoma, A549 lung, MCF7 breast, and HT29 colon cancer cells [106], as well as PS derivative ANK-199-induces autophagic cell death in cisplatin-resistant human oral cancer cells [107], implying that, although autophagy is clearly induced by PS, the mechanisms underlying how PS inhibits tumor progression through autophagy regulation remains ambiguous and warrants further investigation.

### 4.3. Regulation of miRNA Profiles

It is now widely accepted that, like proteins, non-coding miRs are able to alter the intracellular properties and determine the outcomes of tumor progression [120,121]. A recent study indicates that PS down regulates miR-17, -20a, -106a, and -106b while increasing PTEN levels and apoptosis in prostate cancer cells, in that PS reverses PTEN by regulating these miRs [108]. In endometrial cancer (EC), PS significantly suppresses miR663b that plays an oncogenic role by upregulating pro-survival Bcl-2 and reducing apoptosis in EC cells [109]. Indeed, high miR663b expression is correlated with distant metastasis and advanced tumor grading in EC patients [109]. Downregulation of the miR-663b/Bcl-2 pathway may thus serve a new strategy for apoptosis induction by PS.

### 4.4. Inhibiting the Function of Growth Receptors

The AKT and ERK pathway has been demonstrated to be required for androgen receptor (AR) activation in prostate cancer, in which PS-ITC (isothiocyanate) conjugate inhibits AKT, ERK, AR, and their downstream targets, ultimately leading to apoptosis and growth arrest in prostate cancer cells [110]. PS also induces apoptosis in breast cancer cells through deactivation of ER-α36-mediated MAPK/ERK and PI3K/AKT signaling pathways [111]. Our previous study has investigated the chemopreventive effects of PS in urethane-induced murine lung tumors, where PS inhibits epidermal growth factor receptor (EGFR) and its downstream pathways to induce apoptosis and autophagy and retard cell cycle progression during urethane-induced lung tumorigenesis [112].

### 4.5. Affecting Epigenetic Pathway

DNA methylation by DNA methyltransferase (DNMT) and histone modification by histone deacetylase (HDAC including SIRT) and histone acetyl transferase (HAT) play a major role in cell cycle progression, apoptosis, cell death, and proliferation [113]. PS-induced DNA damage response and apoptosis, which results from SIRT1 suppression [113] and hypermethylated and transcriptionally silenced MAML2, is a coactivator of Notch targets at the enhancer region that promotes cancer progression and metastasis in response to PS treatment [114]. In addition, the metastasis-associated protein 1 (MTA1) is a part of nucleosome remodeling and deacetylation (NuRD) co-repressor complex that mediates gene silencing and is inhibited by PS, thereby increasing p53 acetylation, elevating apoptotic index, and lowering angiogenesis in prostate cancer [115]. These studies deliver a novel insight into epigenetic regulation of oncogenic signals in cancer and provide support for epigenetic-targeting strategies by using PS as an effective anti-apoptosis therapeutic approach against cancer.

## 5. Nonapoptotic Effects of PS against Cancer

In addition to the apoptosis-dependent effects of PS on tumor progression, PS has also been found to exert multiple apoptosis-independent efficacies (Table 2), which impacts anti-metastatic therapeutics to complement the potential disadvantages due to apoptotic effect-driven chemoresistance (Figure 2). More detail descriptions are as follows.

### 5.1. Effects in Senescence Induction

Pro-senescence therapy has recently emerged as a novel approach to treat cancers [119]. Previous studies have suggested that therapy-induced senescence can be achieved at far lower chemotherapeutic doses than those required to induce apoptosis, thus reducing the side effects of anticancer therapy [122]. Recently, we further demonstrate a novel anticancer effect of PS by inhibiting the human telomerase reverse transcriptase (hTERT) enzyme activity and protein expression, which results in the subsequent induction of DNA damage, activation of ATM/Chk2 and p53, S phase arrest, and senescence in lung cancer cells [119]. Taking these findings into consideration, the possibility of adopting PS-induced senescence as an alternative strategy to fight against cancer and metastasis may be a novel approach and is worth further investigation.

### 5.2. Effects against EMT

The nonapoptotic and pro-inflammatory effects of Fas-induced EMT [33] has been reported to be highly associated with tumor invasion and metastasis [138] and serves as a target for PS treatment, leading to decrease ERK1/2- and GSK3β/β-catenin-mediated pathways in triple-negative breast cancer (TNBC) cells [123]. Similarly, in in vitro and in vivo experiments, PS has been used to reduce the Fas-associated death domain-mediated FAK signaling [124] that leads to the reversal of EMT and suppresses tumor growth, migration, invasion, and metastasis in TNBC-bearing NOD/SCID mice [108]. Furthermore, PKC-dependent signaling has been known to associate with Fas-induced nonapoptotic and pro-inflammatory effects [32,125]. In line with this concept, it has been found that PS is able to inhibit the PKC activator 12-*O*-tetradecanoylphorbol 13-acetate (TPA)-induced cell motility and metastasis of hepatocellular carcinoma cells [126]. Taken together, PS against Fas-induced EMT could potentially be an alternative strategy in cancer therapy.

### 5.3. Effects against Inflammation

Targeting pro-inflammatory and pro-angiogenesis factors and cytokines may effectively prevent tumor cells from malignantly evolving [139]. IL-1β, TNFα, iNOS, and COX-2 are major players involved in a series of ECM remodeling within pro-inflammatory microenvironments [140,141]. Signaling triggered by the induced expression of selectins, VCAM-1, or intercellular adhesion molecule-1 (ICAM-1) leads to the adhesion and infiltration of inflammatory cells [142]. Indeed, the anti-inflammatory effects of PS to lower mRNA levels of IL-1β, TNFα, iNOS, and COX-2 in HT-29 colon cancer cells have been identified to impact therapeutic strategies against cancer progression [127]. These findings are in good harmony with results coming from a TNFα-induced coculture pro-inflammatory model of 3T3-L1 adipocytes and RAW 264.7 macrophages in which PS significantly decreases the expression of COX-2, iNOS, IL-6, and IL-1β. Additionally, PS is able to penetrate the blood brain barrier to act on TNFα, leading to the inactivation of NF-κB-targeted c-Met in brain-metastatic tumor tissues [128]. Accordingly, the suppressive role of PS in NF-κB signaling pathway has been found to lead to the inhibitory effect in the TNFα-induced expression of VCAM-1 and ICAM-1 in vascular smooth muscle cells [143]. In addition, the anti-inflammatory effects of PS on TNFα involve the pro-inflammatory endoplasmic reticulum stress (ERS) signaling in vascular endothelial cells [144].

### 5.4. Effects against Cell Migration and Invasion

It is well known that EMT-promoted cell migratory and invasive activities facilitate tumor cells to disseminate from primary tissues to distant organs for the establishment of secondary metastases [145]. PS is expected to suppress tumor metastasis as it potently inhibits human lung adenocarcinoma cell migration ability [29]. Moreover, PS suppresses the p38 kinase-mediated aggressive and invasive phenotype of MCF-7 human breast carcinoma cells by reducing MMP-9 activity and growth inhibition [129]. These findings are supported by the evidence that PS inhibits migratory and invasive potentials of triple-negative MDA-MB-231 and Hs578t cells due to overexpression of E-cadherin, decreased expression of Snail, Slug, vimentin, and ZEB1, and down-regulation of MMP-9 [108]. It has been reported that PS suppresses tumor cell migration and invasion by blocking the Rac1/WAVE/Arp2/3 pathway [130]. Furthermore, PS significantly suppresses EMT- and MMP-9-mediated invasion, migration, and metastasis of human hepatoma cells by blocking TPA-induced PI3K/Akt and protein kinase C that are upstream of NF-κB and AP-1 [126]. The anti-EMT effects of PS may also be attributed to the inhibition of IAP-mediated NF-κB activation in an apoptosis-independent manner [71,108].

### 5.5. Effects against Cancer Stemness

CSCs with the ability to differentiate into all cell types found in cancer samples are highly resistance to chemotherapeutic agents and their high metastatic capacity have been considered the important reason for cancer recurrence [39]. PS possesses anticancer activity [39,131,132] through suppressing Wnt/β-catenin-dependent CD133+ stem cell properties in lung cancer [133], glioblastoma [134], hepatocellular carcinoma [135], and breast cancer [39]. Consistently, PS prevents tumor sphere formation, reduces stemness gene expression, and suppresses invasion and migration abilities of CD133+ Mahlavu CSCs, besides its anti-apoptotic effect [135]. Moreover, PS is effectively involved in the suppression of CSC development and thus metastatic potential by impeding the recruitment of type 2 tumor-associated macrophages (M2) via altering the EMT-associated NF-κB/miR488 circuit. [131]. In line with these findings, PS has been found to prohibit macrophage polarization and lung cancer cell stemness by lowering the multifaceted oncoprotein (MUC1), NF-κB, and β-catenin expression [133].

### 5.6. Effects against PolyFN Assembly on CTCs

In addition to both cytotoxic and surgical approaches, effective inhibition of metastases of post-surgical CTCs [9] may alternatively be an ideal anti-cancer adjuvant strategy. PolyFN assembly on tumor cell surfaces has been known to mediate the binding of metastatic CTCs to endothelial cells, leading to the vascular arrest and metastasis of CTCs in the lungs [46,47,48,146]. PS effectively impedes metastasis of suspended Lewis lung carcinoma (LLC) cells in the lungs in an apoptosis-independent manner [49]. Mechanistically, activation of AKT leading to suppressed ERK is responsible for the inhibitory effect of PS on LLC metastasis in the lungs as evidenced by the reversal of PS-mediated metastatic inhibition upon treatment of the PI3K inhibitor LY294002 in reducing pAKT and re-activating pERK, which are then overturned by the treatment with both LY294002 and the ERK inhibitor, suggesting that PS suppressed AKT/ERK-regulated polyFN assembly on suspended LLC cells and pulmonary metastasis [49]. The seemingly paradoxical effects of PS, in that it, on one hand, inactivates PI3K/AKT signaling to induce apoptosis of adherent tumor cells [6] and, on the other hand, activates PI3K/AKT signaling to suppress polyFN assembly on CTCs [49], can be reconciled by the fact that extracellular microenvironments between adherent tumor cells and CTCs are entirely distinct [9]. Understandably, such apparent different microenvironments expectedly render totally distinct AKT-dependent signaling pathways in the tumor cells in response to PS treatment due to the well-known multi-functionalities of AKT [136].

### 5.7. Effects against the Diminished Circulating miR-17-92 Cluster

The metastatic-suppressive roles of the circulating miR-17-92 cluster [63,64] have been demonstrated [65,147]. The miR-17-92 cluster in prostate cancer has been reported to be a downstream player of PS [137]. The tumor suppressive role of PS in inactivation of ERK pathway can be attributed to effects of EMT, in which FN servers as a mesenchymal marker and one of HIF-1α target genes [123,148]. Coincidently, ERK phosphorylation (pERK) that promotes polyFN assembly on blood-borne lung tumor cells has been reported to be suppressed by PS, leading to a reduced lung metastasis [49]. These results suggest that PS may reduce metastasis by elevating the circulating miR-17-92 cluster likely is contained in the form of exosomes [65] and thus inhibiting ERK-dependent polyFN assembly on metastatic CTCs. In line with this idea, ectopic overexpression of miR-20b, a member of miR-17-92, suppresses the TGFβ-induced ERK-dependent proliferation and invasion of papillary thyroid carcinoma [108,149]. PTEN is another target molecule of miR-17-92 [150,151] to consequently terminate the PI3K/AKT signaling [152]. It is worth noting that PS potently activates the AKT-mediated survival signal, followed by an apparent suppression of ERK-dependent polyFN assembly on suspended CTCs and pulmonary metastasis [49]. These results suggest that a reduction of PTEN by the elevated miR-17-92 in the CTCs is responsible for AKT activation by PS, leading to the ERK inactivation. Indeed, PTEN has been found upregulated in CTCs of malignant melanoma and prostate patients with lower survival rates [153,154]. Since miR-17 has the abilities to activate the PI3K/AKT pathway [151] and expression of FN is repressed in cells and tissues expressing miR-17 [66], it is reasonable to expect and warrant further investigations that PS elevates the circulating miR-17 that inhibits PTEN activity to reduce FN expression by activating PI3K/AKT pathway that leads to ERK inactivation.

## 6. Potential Prevention and Treatment of Various Types of Cancer by PS

In this section, we will review our current understanding of the potency of PS in preventing and treating various types of cancer. The possible underlying mechanisms for PS-modulated anti-metastatic effects are also discussed and summarized in Table 3.

### 6.1. Lung Cancer

Assembly of polyFN on circulating breast and lung cancer cells is prerequisite for metastasis in the lungs, suggesting that polyFN may be targeted by drugs to fight against cancer metastasis [48]. In light of this possibility, we have recently demonstrated that both PS and FN-silencing significantly reduce polyFN assembly and lung metastasis of suspended LLC cells in an apoptosis-independent manner. Oral administration of PS sufficiently and significantly prevents lung-colonization and -metastasis of LLC cells (apoptosis independent) and reduces the already established tumor growth in the mouse lungs (apoptosis dependent) [49]. Induction of both apoptosis and autophagy leading to an inhibition of lung tumor growth and metastasis was found in our previous study regarding the chemopreventive effects of PS on urethane-induced lung carcinogenesis at doses of 50 or 250 mg/kg [112]. For a new anti-NSCLC treatment, PS has been employed to be intraperitoneally administered every day for 50 mg/kg in athymic nude mice xenografts with the subcutaneously inoculated NSCLC PC9 cells. Tumor sizes have been found to be significantly reduced due to an enhancement of the ERS signaling. Meanwhile, the migratory and adhesive abilities and intracellular glutathione (GSH) level are inhibited and ROS generation, caspase 3 activity, and mitochondrial membrane depolarization are enhanced [29].

### 6.2. Breast Cancer

Recently, PS has been reported to suppress tumor growth and metastasis in MDA-MB-231-bearing NOD/SCID mice by reducing Src/Fak signaling [111]. Furthermore, PS, a blood brain barrier penetrable natural compound, has been found to attenuate the metastatic growth of breast cancer cells in the brain [155]. Additionally, PS suppresses the formation of metastatic breast cancer stem cells within tumor microenvironment via modulating EMT associated signaling pathways, especially the NF-κB/microRNA 448 circuit [131]. The carcinogenic pathways inhibited by PS treatment are similar to pathways altered by blueberry juice, making it plausible that PS is the compound responsible for the anticarcinogenic effects of blueberry treatment in breast cancer [163]. PS also produces a synergistic inhibitory effect when combined with the chemotherapy drug Tamoxifen, demonstrating clinical potential in the treatment of breast cancer [164].

### 6.3. Prostate Cancer

The functional relevance of metastasis-associated protein 1 (MTA1) in promoting human prostate tumor growth, invasion, angiogenesis and metastasis has been highlighted both in vitro and in xenograft animal models [115,165]. The inhibition of MTA1 in inducing c-Myc/Akt-mediated inflammation and EMT by dietary PS resulting in decreased proliferation and angiogenesis has also been demonstrated in prostate-specific PTEN-null (Pb-Cre^+^; PTEN^f/f^; Rosa26^Luc/+^) C57BL/6J mouse model [156,157]. In addition, PS increases the levels of MTA1-mediated p53 acetylation in an orthotopic prostate cancer xenograft animal model, from which PS significantly inhibits tumor growth, progression, local invasion and spontaneous metastasis [115]. These studies offer a pre-clinical proof for PS as a promising chemopreventive and therapeutic agent to curb prostate cancer. In a combinatory therapeutic strategy, dietary administration of PS has been found to sensitize tumor cells to histone deacetylases (HDAC) 1 and 2 inhibitor SAHA in a prostate-specific PTEN-null mouse model, in which tumor growth is inhibited and tumor progression is declined [158].

### 6.4. Hepatocellular Melanoma and Myeloma Cancers

PS has been shown to significantly suppress TPA-induced PI3K-AKT-NF-κB signaling axis-mediated invasion, migration and metastasis of hepatocellular carcinoma Hep-G2 cells through down-regulation of MMP-9 both in vitro and in vivo [126]. Both in vitro and in vivo studies have been conducted to reveal the anti-metastatic potency of PS against highly malignant B16 melanoma F10 cells (B16M-F10) by inhibiting VCAM-1 expression in the hepatic sinusoidal endothelium and Bcl-2 expression in metastatic cells. Intravenous administration of PS (20 mg/kg per day) to mice inhibits 73% metastatic growth of B16M-F10 cell in the liver, a common site for metastasis development [159]. For evaluating anti-multiple myeloma effect of PS, H929 cells has been injected into the flanks of female NOD/SCID mice and treated with PS for 14 days via intraperitoneal injection. The results show that, compared to control animals, tumor volumes are significantly decreased in the PS-treated mice, indicating the potent anti-myeloma effect of PS in vivo [160].

### 6.5. Colon Cancer and Pancreatic Cancer

Dietary administration of PS reduces tumor multiplicity of the azoxymethane (AOM)-induced colon cancer animal model. Tumor incidence of AOM-induced rats is decreased from 87.5% to 67.8% upon PS treatment [132]. Similarly, the tumor multiplicities of the AOM-induced colon cancer in male BALB/c and ICR mice are significantly decreased after dietary administration of PS via induction of apoptosis [91,161,162]. When PS is orally administered at 100 μg/kg/day or 500 μg/kg/day to nude mice bearing human pancreatic tumor for eight weeks, tumor growth rates are significantly inhibited in a dose-dependent manner without causing any acute toxicity [104].

## 7. Future Perspective

PS clearly has an inhibitory effect on various types of cancer through oral, intraperitoneal, and intravenous administration in mouse and rat models. Based on these efforts, we are on the right track moving toward discoveries of novel preventive and therapeutic strategies. It is worth noting that, for the convenience and safety reasons, these findings encourage the future development of PS into natural food products that warrant further investigation and trials as an alternative medicine in cancer patients. Here we summarize the new directions that may lead the study of PS to the bright future on better treatment of cancer.

### 7.1. Potential Drug Packing and Delivery System for PS

Ingenious strategy for drug packing and delivery is needed to improve the administration of PS in cancer prevention and treatment. Various methods of drug packing for delivery have been developed. For examples, gold nanoparticles (GNPs) and liposomes have been used as the model systems to construct a hybrid system and investigate its performance for the tumor therapy of Paclitaxel [166]. Fabricating devices for drug delivery by using the Poly(lactic-*co*-glycolic) acid (PLGA), which has been approved in clinical use by the US Food and Drug Administration (FDA), has attracted attentions as a base material for biomedical applications [167,168]. PS may be packed into these nanoparticles in elevating drug delivery efficacies. Similarly, magnetic guidance employing an extracorporeal magnetic field on the biological target during the injection of a magnetically responsive nanocarrier has improved drug accumulation and been demonstrated to have great potential in experimental cancer therapy [169]. Moreover, the quantum dots which allow their incorporation within virtually any nanoparticle-based drug delivery vehicle due to the features of small size and versatile surface chemistry with minimal effect on overall characteristics offer excellent optical properties for real-time monitoring of drug delivery [170,171]. Another material which can be considered as a drug delivery device for PS is graphenes, a two-dimensional monolayer of sp2 hybridized carbon arranged in a hexagonal packed structure [172]. The exploitation of exosomes as drug delivery vehicles offers important advantages in that exosomes are non-immunogenic in nature due to similar composition as body’s own cells [173]. All of these drug packaging and delivery methodologies can hopefully be applied in the future development of PS-based anti-cancer therapeutics.

### 7.2. FN-Targeting and PS Combined Therapy

Disrupting signaling pathways leading to inhibition of biosynthesis of FN or polyFN assembly on CTCs may contribute to unprecedented discoveries of novel therapeutic approaches to cancer metastatic prevention [174]. A number of FN-targeting ligands have been developed for cancer therapies [175]. Dietary PS has been experimentally employed to suppress polyFN assembly on suspended LLC cells and pulmonary metastasis through AKT-ERK regulatory signaling axis [49]. Thus, FN is expected to be used as an indicator to evaluate the efficacies of orally administered PS in the future clinical trials. Additionally, FN isoforms comprising the EDA or EDB domains also known as oncofetal forms of FN [176] may be used as cancer vaccines to prevent certain cancer types [177]. Co-treatment with PS and FN vaccination (or any FN-targeting compounds) could be a potential future direction of combinatory therapy.

### 7.3. PS-Enhanced Immunotherapy

Cancer immunotherapies employing T-cell-associated checkpoint inhibitor, e.g., pembrolizumab and ipilimumab, currently approved by U.S. FDA and European Medicines Agency, inhibit the programmed death-1 (PD-1) pathway and the cytotoxic T-lymphocyte-associated protein 4 (CTLA4), respectively, have prevailed in recent cancer therapeutic policies [178]. Interestingly, PS has been used as an adjuvant to locally improve inhibition of HIV-infection in resting CD4 T cells [179]. PTEN loss in tumor cells may be a common ground responsible for effects of checkpoint inhibitors on cancer immunotherapies and PS on HIV infection in T cells and tumor progression as aforementioned in Section 4.2.7 [180]. It is worth investigating that combining checkpoint inhibitors and oral administration of PS as natural food products may be established to enhance patients’ immunity to fight with cancer malignancy, likely opening a brand new direction to pharmaceutical cancer therapy.

### 7.4. PS Modification

PS, a double-methylated version of resveratrol, is a stilbenoid and a structural analog of resveratrol. This modification makes PS more stable in vivo and exhibit higher bioavailability than resveratrol [97]. Resveratrol has another modification called pterostilbene carboxaldehyde thiosemicarbazone (PTERC-T) [97]. PTERC-T has been found to inhibit angiogenesis induced by sex steroids, particularly 17β-Estradiol female (E2), which involves thrombospondins-1. Thus, PTERC-T has been considered as a novel drug targeting angiogenesis-related diseases, including tumor progression and metastasis [181]. These results shed some light on the potential possibility of discovering unique chemical modifications on PS to exert stronger anti-cancer and anti-metastatic efficacies and reduced side effects caused by any non-specificity of PS on regular homeostasis and physiology in the future.

### 7.5. Future Prospect of PS in Clinical Approach

Recent studies in human and in animal models have investigated the safety profile of PS and suggest that PS does not have significant toxic effects. A previous clinical trial indicates that PS is generally safe for use in humans at doses up to 250 mg per day [182]. There are no adverse drug reactions (ADRs) as indicated by hepatic, renal, or glucose markers, and no statistically significant self-reported or major ADRs [182]. Another clinical trial points out that employing PS (pTeroPure) at 250 mg/day in adults attains safe status [183]. In addition, a large number of studies have shown the great potential of PS on chemoprevention and anti-cancer effects. These studies demonstrate that PS with great anti-cancer potential seems to be well-tolerated and safe in humans. However, little is clinically known about the pharmacokinetics, bioavailability, metabolism, and anti-cancer effects. This suggests that further clinical investigations are needed to address the potentially multiple biomedical applications of PS, any undesirable side effects after prolonged administration, and the efficacy of anti-cancer effects of PS so as to portray the comprehensive safety aspects related to its clinical usages.

## 8. Conclusions

While cancer patients receiving cytotoxic therapies clinically suffer unwanted side effects, drug-resistant relapses, and metastatic recurrence, the multi-functionalities of PS, one of potential alternative medicines, may complement the current therapeutic strategies and reduce therapy-derived disadvantages. Reviewing the updated literature, we conclude that PS has inhibitory effects on almost every cellular event that promotes tumor progression toward metastasis in apoptosis-dependent as well as apoptosis-independent manners. Most importantly, PS, upon oral administration, is able to simultaneously prevent colonization of CTCs and diminish the already established secondary tumor masses in distant organs, greatly lowering the risks of patients for metastatic recurrence due to drug-resistance. Accordingly, PS is an ideal choice of alternative medicine to be administered in the diet as nutritional supplements. In the future, the potency and drug ability of PS can even be improved by chemical modification or conjugation with nanoparticles that help pack and deliver PS into tumor tissues. Moreover, combining with T-cell-associated checkpoint inhibitors, PS may greatly enhance the immunotherapeutic efficacies. Therefore, employing PS as a complementary and alternative medicine may be a prevailing therapeutic strategy against cancer malignancy.

## Figures and Tables

**Table 1:** Summary of pro-apoptotic effects of PS against cancer.

Gene/Phenomena	Effect/Regulation	Pathway	Tumor Types/Model *	Reference
**Enhancement of endoplasmic reticulum stress (ERS) signaling**				
IRE-1, p53, ATF4, p-PERK, CHOP9	up	ERS	NSCLC	[29]
Bcl-2	down	ERS	HEC	[100]
PUMA, caspases 9, caspases12	up	ERS	HEC	[100]
cell proliferation	down	ERS	bladder cancer	[101]
ROS generation	up	ERS-ROS	breast cancer prostate cancer	[91,102,103,104]
antioxidant activity	up	ERS-ROS	pancreatic cancere sophageal cancer colon cancer	[91,102,103,104]
**Induction of autophagy**				
chemotherapeutic effect	up	autophagy	lung cancer	[105]
enhance apoptosis	up	apoptosis	bladder cancer	[101]
cancer cell growth	down	apoptosis	A375, A549, MCF7, HT29	[106]
autophagic cell death	up	autophagy	human oral cancer	[107]
**Regulation of miRNA profiles**				
miR-17, miR-20a, miR-106a, miR-106b	down	apoptosis	prostate cancer	[108]
PTEN	up	apoptosis	prostate cancer	[108]
miR663b	down	apoptosis/Bcl-2	EC	[109]
**Inhibiting the function of growth receptors**				
AKT, ERK, AR	down	AKT/ERK	prostate cancer	[110]
ER-α36	down	MAPK/ERK, PI3K/AKT	breast cancer	[111]
EGFR	down	apoptosis, autophagy	murine lung tumors	[112]
**Affecting epigenetic pathway**				
SIRT1	down	DNA damage response, apoptosis	breast cancer	[113]
MAML2	down	DNA damage response, apoptosis	breast cancer	[114]
MTA1	down	p53 acetylation	prostate cancer	[115]

* NSCLC, Non-Small Cell Lung Cancer; HEC, human esophageal cancer; EC, endometrial cancer.

**Table 2:** Summary of nonapoptotic effects of PS against cancer.

Gene/Phenomena	Effect/Regulation	Pathway	Tumor Types/Model *	Reference
**Effects in senescence induction**				
hTERT	down	senescence	lung cancer	[119]
ATM/Chk2	up	senescence	lung cancer	[119]
p53	up	senescence	lung cancer	[119]
**Effects against EMT**				
Fas-induced EMT	down	ERK1/2, GSK3β/β-catenin	TNBC	[123]
Fas-associated death domain	down	FAK signaling	TNBC	[108,124]
TPA	down	PKC-dependent signaling	HCC	[32,125,126]
**Effects against inflammation**				
IL-1β, TNFα, iNOS, and COX-2	down	inflammatory microenvironments	HT-29	[127]
COX-2, iNOS, IL-6, and IL-1β	down	inflammatory	3T3-L1	[128]
Effects against cell migration and invasion				
cell migration	down	cell migration ability	human lung adenocarcinoma	[29]
p38 kinase	down	MMP-9 activity	MCF-7	[129]
MMP-9	down	migratory and invasive	MDA-MB-231, Hs578t	[108]
cell migration and invasion	down	Rac1/WAVE/Arp2/3 pathway	MDA-MB-231	[130]
TPA-induced PI3K/Akt and protein kinase C	down	EMT- and MMP-9-mediated invasion, migration and metastasis	human hepatoma cell	[126]
IAP	down	NF-κB activation	malignant pancreas cell	[71,108]
**Effects against cancer stemness**				
CD133	down	Wnt/β-catenin	lung cancer, glioblastoma, HCC, breast cancer	[39,131,132], [133], [134], [135]
M2 macrophage	down	EMT-associated NF-κB/miR488	breast cancer	[135]
MUC1, NF-κB, β-catenin	down	macrophage polarization and lung cancer cell stemness	lung cancer	[133]
Effects against polyFN assembly on CTCs				
ERK	down	PI3K/AKT signaling	LLC	[6,49,136]
**Effects against the diminished circulating miR-17-92 cluster**				
miR-17-92	up	PTEN	prostate cancer	[137]

* TNBC, triple-negative breast cancer; LLC, Lewis lung carcinoma; HCC, hepatocellular carcinoma cells.

**Table 3:** Summary of potential prevention and treatment of various types of cancer by PS.

Administration	Doses	Animal Models	Reference
**Lung cancer**			
oral	5 mg/kg	C57BL6 mice	[49]
oral	50 or 250 mg/kg	A/J mice	[112]
intraperitoneally	50 mg/kg	nude mice	[29]
**Breast cancer**			
oral	56 mg/kg	nude mice	[111]
intraperitoneally	30 mg/kg	nude mice	[155]
intraperitoneally	5 mg/kg	NOD/SCID mice	[131]
**Prostate cancer**			
oral	100 mg/kg diet	PTEN-heterozygous mice	[156,157]
intraperitoneally	50 mg/kg	orthotopic PCa xenograft	[115]
oral	10 mg/kg	PTEN-null mice	[158]
**Hepatocellular melanoma and myeloma cancer**			
Intravenous	20 mg/kg		[159]
intraperitoneal	50 mg/kg	NOD/SCID mice	[160]
**Colon cancer and pancreatic cancer**			
oral	40 ppm	F344 rats	[132]
oral	50 ppm	BALB/c mice	[91,161]
oral	40 ppm	F344 rats	[162]
oral	100 μg/kg/day or 500 μg/kg/day		[104]
